# Integrative Analysis of the Expression Levels and Prognostic Values for NEK Family Members in Breast Cancer

**DOI:** 10.3389/fgene.2022.798170

**Published:** 2022-03-16

**Authors:** Wen-Liang Gao, Lei Niu, Wei-Ling Chen, Yong-Qu Zhang, Wen-He Huang

**Affiliations:** ^1^ Department of Breast-Thyroid-Surgery and Cancer Research Center, Xiang’an Hospital of Xiamen University, Xiamen, China; ^2^ Xiamen Research Center of Clinical Medicine in Breast and Thyroid Cancers, Xiamen, China; ^3^ Xiamen Key Laboratory of Endocrine-Related Cancer Precision Medicine, Xiamen, China

**Keywords:** breast cancer, NEK family, biomarkers, prognosis values, bioinformatics, immune system

## Abstract

**Background:** In the latest rankings, breast cancer ranks first in incidence and fifth in mortality among female malignancies worldwide. Early diagnosis and treatment can improve the prognosis and prolong the survival of breast cancer (BC) patients. The NIMA-related kinase (NEK), a group of serine/threonine kinase, is a large and conserved gene family that includes NEK1–NEK11. The NEK plays a pivotal role in the cell cycle and microtubule formation. However, an integrative analysis of the effect and prognosis value of NEK family members on BC patients is still lacking.

**Methods:** In this study, the expression profiles of NEK family members in BC and its subgroups were analyzed using UALCAN, GEPIA2, and Human Protein Atlas datasets. The prognostic values of NEK family members in BC were evaluated using the Kaplan–Meier plotter. Co-expression profiles and genetic alterations of NEK family members were analyzed using the cBioPortal database. The function and pathway enrichment analysis of the NEK family were performed using the WebGestalt database. The correlation analysis of the NEK family and immune cell infiltration in BC was conducted using the TIMER 2.0 database.

**Results:** In this study, we compared and analyzed the prognosis values of the NEKs. We found that NEK9 was highly expressed in normal breast tissues than BC, and NEK2, NEK6, and NEK11 were significantly highly expressed in BC than adjacent normal tissues. Interestingly, the expression levels of NEK2, NEK6, and NEK10 were not only remarkably correlated with the tumor stage but also with the molecular subtype. Through multilevel research, we found that high expression levels of NEK1, NEK3, NEK8, NEK9, NEK10, and NEK11 suggested a better prognosis value in BC, while high expression levels of NEK2 and NEK6 suggested a poor prognosis value in BC.

**Conclusion:** Our studies show the prognosis values of the NEKs in BC. Thus, we suggest that NEKs may be regarded as novel biomarkers for predicting potential prognosis values and potential therapeutic targets of BC patients.

## Introduction

Breast cancer (BC) is the most common malignant tumor in women, and its morbidity and mortality rank first. BC has seriously threatened the lives of patients, and the prevention and control situation is severe (Bray et al., 2018). At present, BC clinical treatments mainly include local surgery combined with comprehensive treatment methods such as chemotherapy, radiotherapy, endocrine therapy, and targeted therapy. However, disease-free survival (DFS) and overall survival (OS) of BC patients have not been effectively improved. Studies have shown that about 20% of patients with metastatic BC survive less than 5 years (Sun et al., 2019), which may be due to the lack of specific biomarkers associated with early diagnosis, treatment targets, and the prognosis assessment of BC as well as the fact that the occurrence, development, and metastasis mechanism of BC have not been fully clarified clinically until now. Therefore, the search for BC biomarkers is of great clinical significance for BC diagnosis and treatment.

Protein kinases (PKs) account for 1.7% of all genes in the human genome and play an important role in the signal transduction of eukaryotic cells (Melo-Hanchuk et al., 2020). The regulation of cell cycles by PKs, especially during checkpoints, is an attractive therapeutic target in tumors (Lapenna and Giordano, 2009). The NIMA-related kinase (NEK) plays a crucial role in regulating different aspects of the cell cycle. Originally found in the fungus Aspergillus, NIMA (Never in MitosisA) is a serine/threonine kinase (79 kDa) that is essential to perform mitosis. Subsequently, NIMA has been shown to play a role in all phases of cell division (Peres de Oliveira et al., 2020). In mammals, there are 11 PKs that share 40–50% of the amino acid sequence identity with NIMA in their catalytic domain (Peres de Oliveira et al., 2020), so they are called NEK: NIMA-related kinase (NEK). The 11 PKs are named NEK1–NEK11 (Prosser et al., 2016) and are located in cilia, centrosomes, cell nucleus, cytoplasm, and mitochondria (Melo-Hanchuk et al., 2020). Members of the NEK family are involved in the development and/or progression of several diseases (de Carcer et al., 2007; Malumbres, 2011). Different from normal tissues, the NEK family has high ectopic expression and genovariation in many malignant tumors, leading to the abnormal cell cycle regulation and development of tumors (Joseph et al., 2020). With the development of bioinformatics, it is highly possible and convenient to study the underlying occurrence and development mechanism of BC, explore new targets and therapeutic methods, and improve the survival rate of BC patients.

## Materials and Methods

### Reverse Transcription and PCR Analysis

The protocols of total RNA extraction and purification, cDNA reverse transcription, and RT- PCR were described in previous studies (Dou et al., 2017). All primers used for the RT-PCR analysis are shown in Table 1.

**TABLE 1:** Sequence of real-time PCR primers.

The name of the primer	Primer sequences
NEK1	F: GGC​AGC​TAA​GTG​TGT​GAG​TGG​ATC
	R: TCT​CCT​CGT​TTG​GCT​TTC​TTG​TCT​G
NEK2	F: AGG​TAC​TGA​AGA​GTG​AGC​CGT​ATG​G
	R: TGT​AGC​CAA​GGA​CAG​CAT​GTT​AGT​G
NEK3	F: GCA​GCA​CGA​CAA​CAT​TAT​TGC​CTA​C
	R: CCA​CAC​CAC​CAT​CTC​TTC​CTC​AAA​C
NEK4	F: GAC​TTC​TTC​ACT​GCC​TGC​CTG​AC
	R: CAA​TGG​TGG​GCT​GGC​TGA​TGT​C
NEK5	F: ACA​GTG​TGA​CTA​CCC​ACC​TCT​TCC
	R: TCT​GGT​CGC​TTC​TCT​GGA​TCT​GG
NEK6	F: TGG​ACA​GGA​AGA​CAG​TGG​CTC​TG
	R: GGG​TGG​TTC​AGT​TGC​TTC​AAG​AGG
NEK7	F: TGA​GGA​GGA​GAG​CCC​ACA​AAC​C
	R: GCC​TCG​TTT​CCT​GAC​CGT​AAT​CC
NEK8	F: GTC​TCT​TGA​AGT​CAC​CTG​CCA​GTC
	R: ACA​AAG​CCA​TCA​CTC​ACT​GAA​TCC​C
NEK9	F: AGA​TGA​GGA​GGA​CAC​GGA​CTT​TGA​G
	R: GCC​ATC​CAG​CTC​GCT​TCT​TCA​G
NEK10	F: TTG​TGA​AGG​AGG​GGA​TCT​GGC​TAG
	R: ACC​ATC​ACT​TCG​TCT​GTG​GCA​TTC
NEK11	F: CGA​GCC​GAA​GGA​CAT​ATG​GGA​ATC
	R: TTG​CCT​CAG​TCT​TGC​CAG​ATA​AAC​C

### UALCAN

The UALCAN database (http://ualcan.path.uab.edu/index.html) contains many clinical data for various cancer types, which can be used to query the relative expression of one or several genes and their impact on survival time of cancer patients in various subgroups of tumors and normal tissue samples (Chandrashekar et al., 2017). In this study, we used the UALCAN database to examine the difference in mRNA expression levels of NEKs between BC and healthy breast tissues.

### GEPIA2

The GEPIA2 website (http://gepia2.cancer-pku.cn/) is freely used to analyze the gene expression of 9,736 tumors and 8,587 healthy samples from the TCGA and GTEx databases (Joseph et al., 2020). In this study, the GEPIA2 database was used to verify the expression of NEKs based on the GEO and TCGA databases.

### Human Protein Altas

The Human Protein Atlas (*HPA*, https://www.proteinatlas.org) database contains immunohistochemical expression in normal and cancer tissues (Asplund et al., 2012). In this study, the *HPA* database provided immunohistochemical staining of NEK gene family proteins in normal and BC tissues.

### Kaplan–Meier Plotter

Kaplan–Meier Plotter (http://kmplot.com) is an online database of cancer clinical gene chips published online, which is constructed based on the gene chip and high-throughput expression profile from public databases. We used the Kaplan–Meier plotter to analyze the prognostic correlation of NEK expression levels in BC.

### cBioPortal

The cBioPortal database provides a visual tool for studying and analyzing cancer-related genetic data, including 283 large tumor-related gene expressions and proteomic studies from multiple databases (Gao et al., 2013). We utilized the cBioPortal database to investigate the mutation frequency and form of NEK mutations.

### STRING

STRING 11.0 (https://string-db.org/) is a database of protein interactions to analyze direct and indirect interactions between known and predicted proteins and provide gene ontology (GO) and Kyoto Encyclopedia of Genes and Genomes (KEGG) enrichment results (Szklarczyk et al., 2021). This study analyzed the interaction between NEKs and protein using the STRING 11.0 database with the minimum required interaction score set as 0.05.

### GeneMANIA

GeneMANIA (http://www.genemania.org) is a website that provides genetic information and prioritizes genes for functional analysis with highly accurate prediction algorithms and predicts the relationships between genes and their similar genes (Warde-Farley et al., 2010; Franz et al., 2018). We used the GeneMANIA website to search and classify the interactions between NEKs and their adjacent genes.

### WebGestalt

WebGestalt is a free online site that focuses on enrichment analysis, supports a variety of enrichment analysis algorithms, and covers a detailed database of functional annotations. In this study, GO and KEGG enrichment analysis and functional annotation were conducted for the screened genes by using the WebGestalt database.

### Statistical Analysis

The difference in NEKs mRNA expression was examined by using the *t*-test, Kaplan–Meier survival was tested by using the log-rank test in the UALCAN database, and Pearson correlation analysis was used to analyze the genes co-expressed with NEKs. All database analyses proved that the difference was statistically significant (*p* < 0.05).

## Results

### The mRNAs Expression Levels of NEK1–NEK11 in Breast Cancer Patients and Cell Lines

**FIGURE 1:**
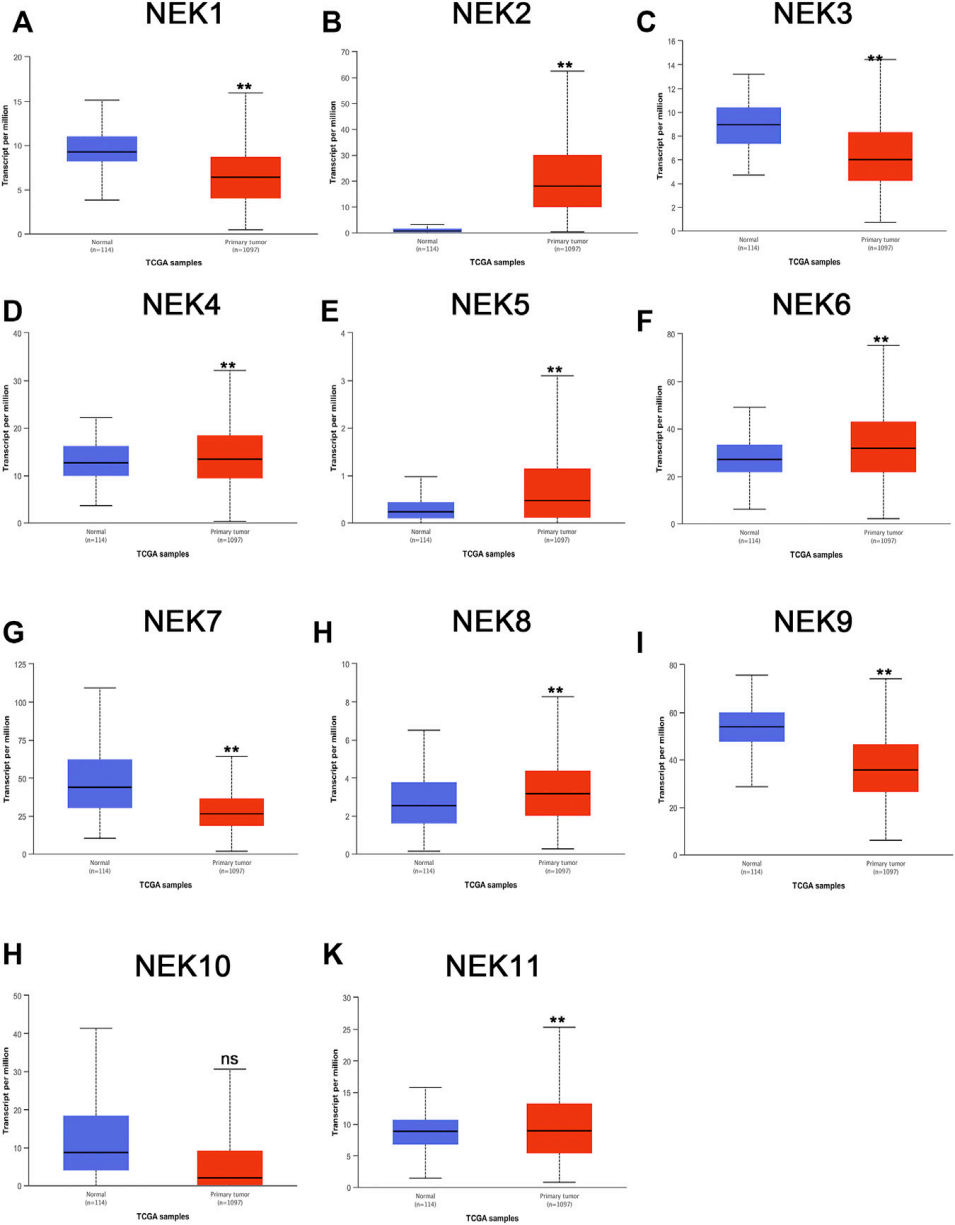
The mRNA expression levels of NEK family members in distinct tumor types. The graphs generated from the UALCAN database show the numbers of datasets with the statistically significant expression level of the NEK family in BC tissues and normal breast tissues. **p < 0.01, ns: no statistically significant **(A–K)** represents NEK family members NEK1–NEK11.

**FIGURE 2:**
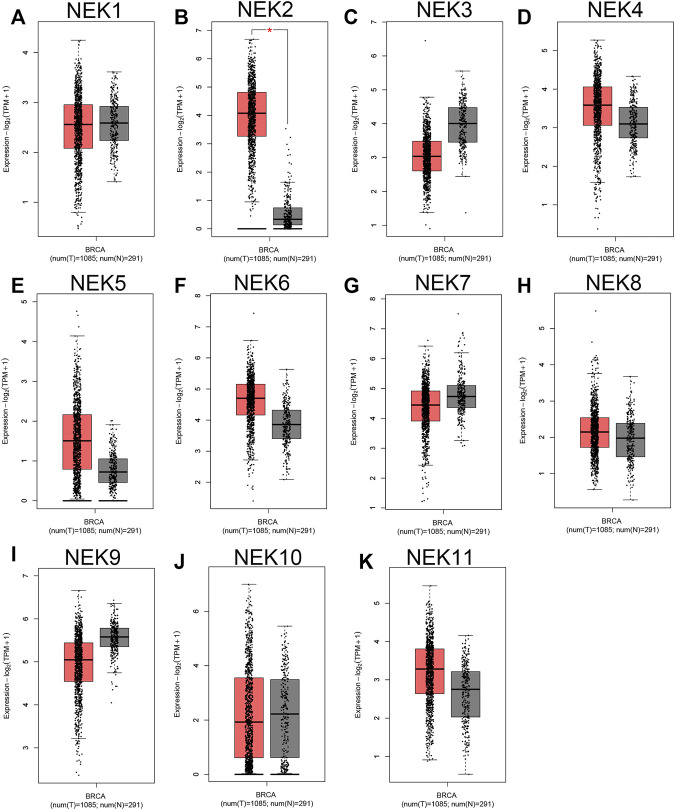
The expression level of the NEK family in BC compared with the normal. The graphs generated from the GEPIA database show the comparison of the NEK family gene expression between BC tissues (BRCA) (*n* = 1,085) and normal breast tissues (*n* = 291), *p < 0.05. Tumor tissues are shown in red, and normal tissues are shown in gray. **(A–K)** represents NEK family members NEK1–NEK11.

**FIGURE 3:**
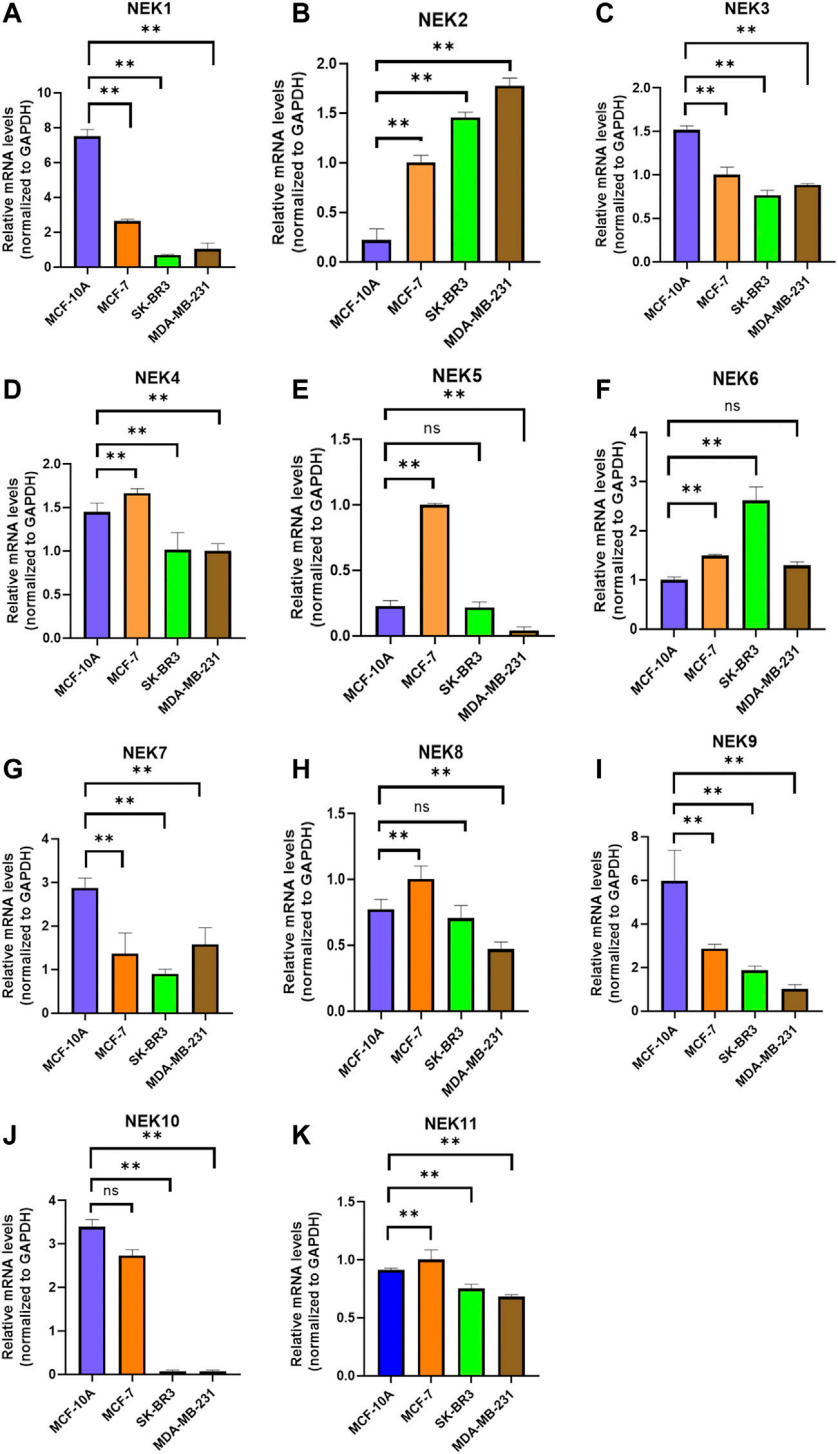
Relative NEK family members’ mRNA levels in breast cancer cell lines quantified by real-time PCR. **(A)** NEK1; **(B)** NEK2; **(C)** NEK3; **(D)** NEK4; **(E)** NEK5; **(F)** NEK6; **(G)** NEK7; **(H)** NEK8; **(I)** NEK9; **(G)** NEK10; and **(K)** NEK11. The experiments were repeated three times. *p < 0.05, **p < 0.01, ***p < 0.001, ns: no statistically significant.

The mRNAs expression of the NEK gene family in BC and normal tissues were investigated by using the UALCAN database. NEK2, NEK4, NEK5, NEK6, NEK8, and NEK11 were highly expressed in BC, and NEK1, NEK3, NEK7, NEK9, and NEK10 were highly expressed in healthy breast tissues (Figure 1). Subsequently, we utilized the GEPIA2 database to contrast the expression levels of NEK1–NEK11 in BC and normal breast tissues again. The results of the GEPIA2 database were roughly the same as those of the UALCAN database (Figure 2). Our study confirmed that the expression level of NEK2 was significantly higher in four kinds of breast cancer than in MCF-10A. The expression level of NEK6 in breast cancer cells (MCF-7 and SK-BR-3) was higher than that in normal breast epithelial cells in MCF-10A, and the expression level of NEK8 and NEK11 was higher than that in normal epithelial cells in MCF-7 (*p* < 0.01). The expression level of other NEK family members is shown in Figure 3. These results suggest that the high expression of NEK2, NEK4, NEK5, NEK6, NEK8, and NEK11 genes may be involved in the development of BC.

### Analysis of Protein Expression of the NIMA-Related Kinase Gene Family in Breast Cancer

**FIGURE 4:**
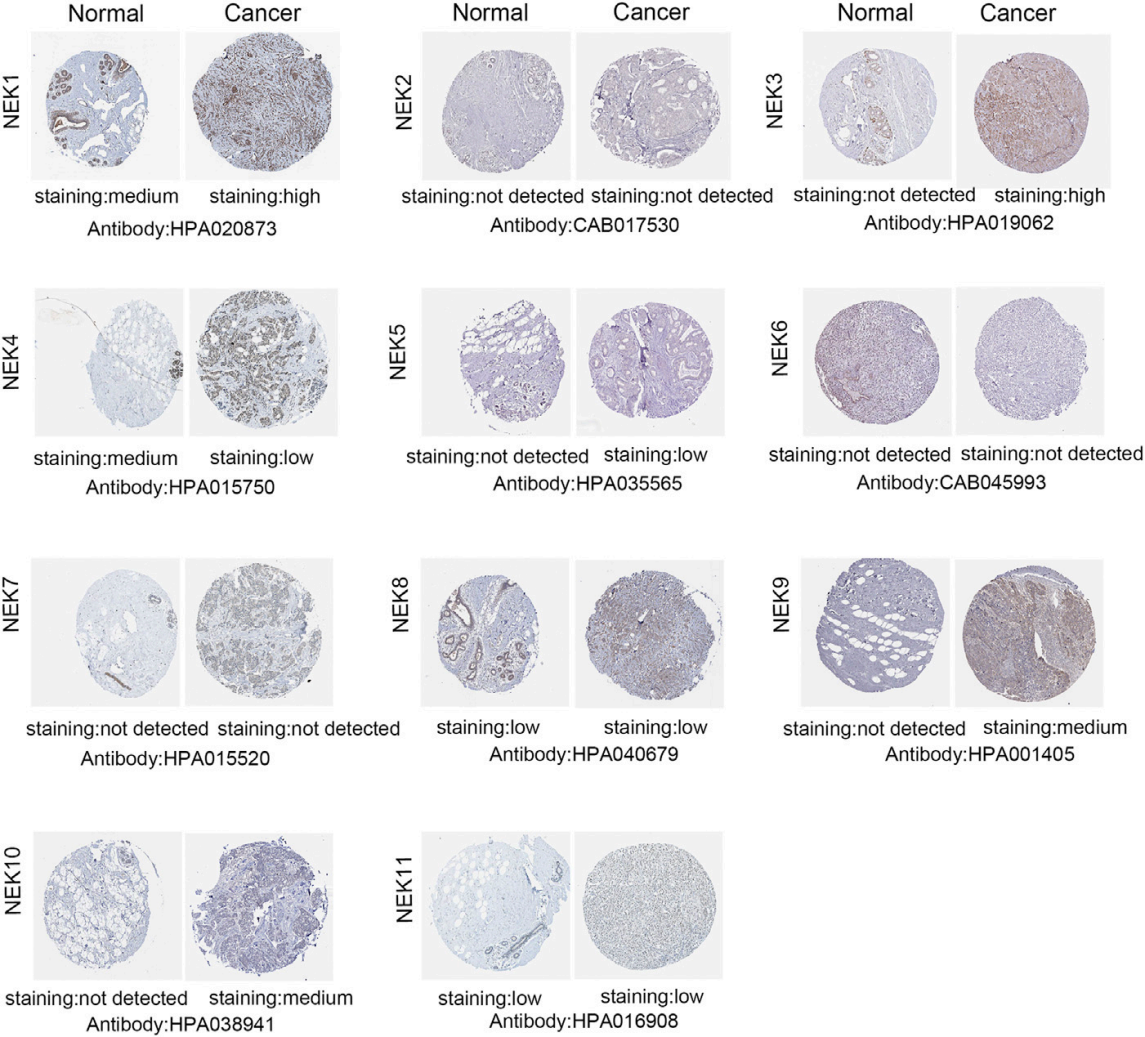
Representative immunohistochemical images of NEKs in normal breast tissues and BC tissues (*HPA* Database) (200 µm).

In order to study the protein level of the NEK gene family in BC tissues, we demonstrated the difference in the expression of NEK family protein in BC and normal breast tissues through the *HPA* database. As demonstrated in Figure 4, the expressions of NEK1, NEK3, NEK5, and NEK9 proteins in BC tissues were higher than those in normal breast tissues. The expression of the NEK4 protein in BC tissues was lower than that in normal breast tissues. NEK2, NEK6, NEK7, NEK8, NEK10, and NEK11 protein expressions were not significantly different between BC tissues and normal breast tissues.

### The Transcription Level of the NIMA-Related Kinase Gene Family Was Significantly Related to the Molecular Subtype and Tumor Stage of Breast Cancer

**FIGURE 5:**
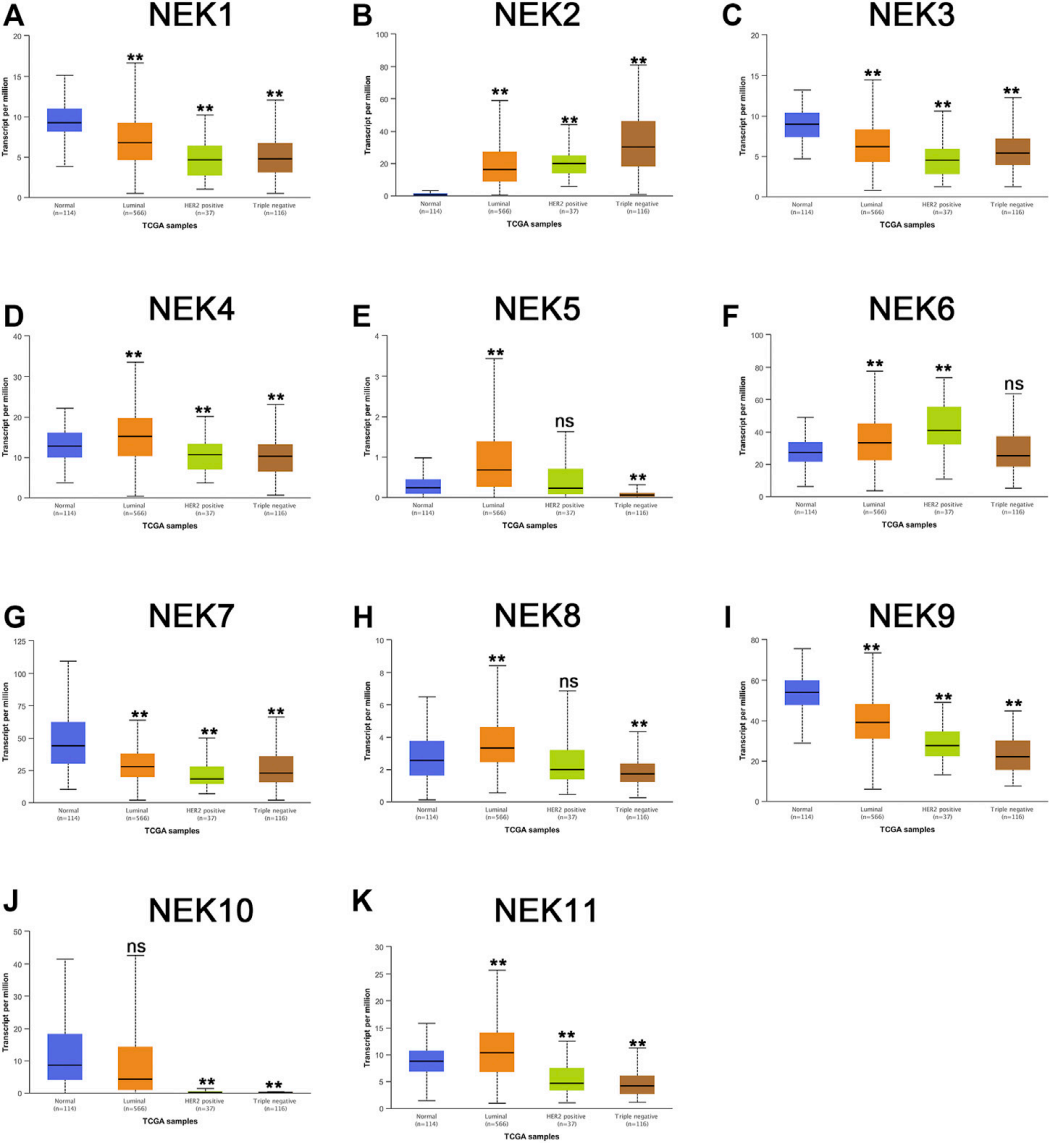
The correlation between the NEK family expression and molecular subtype in BC patients. The graphs produced by using the UALCAN database show the expression of NEK family genes in different molecular subtyping of BC. Normal tissues are shown in red, luminal types are shown in orange, Her2-positive types are shown in green, and triple-negative types are shown in brown. **(A–K)** represents NEK family members NEK1–NEK11. Breast cancer cell lines MCF-7, SK-BR-3, and MDA-MB-231 were compared with the normal epithelial cell MCF-10A, and then Student’ *t* test statistics were conducted. *p < 0.05, **p < 0.01, ns: no statistically significant.

**FIGURE 6:**
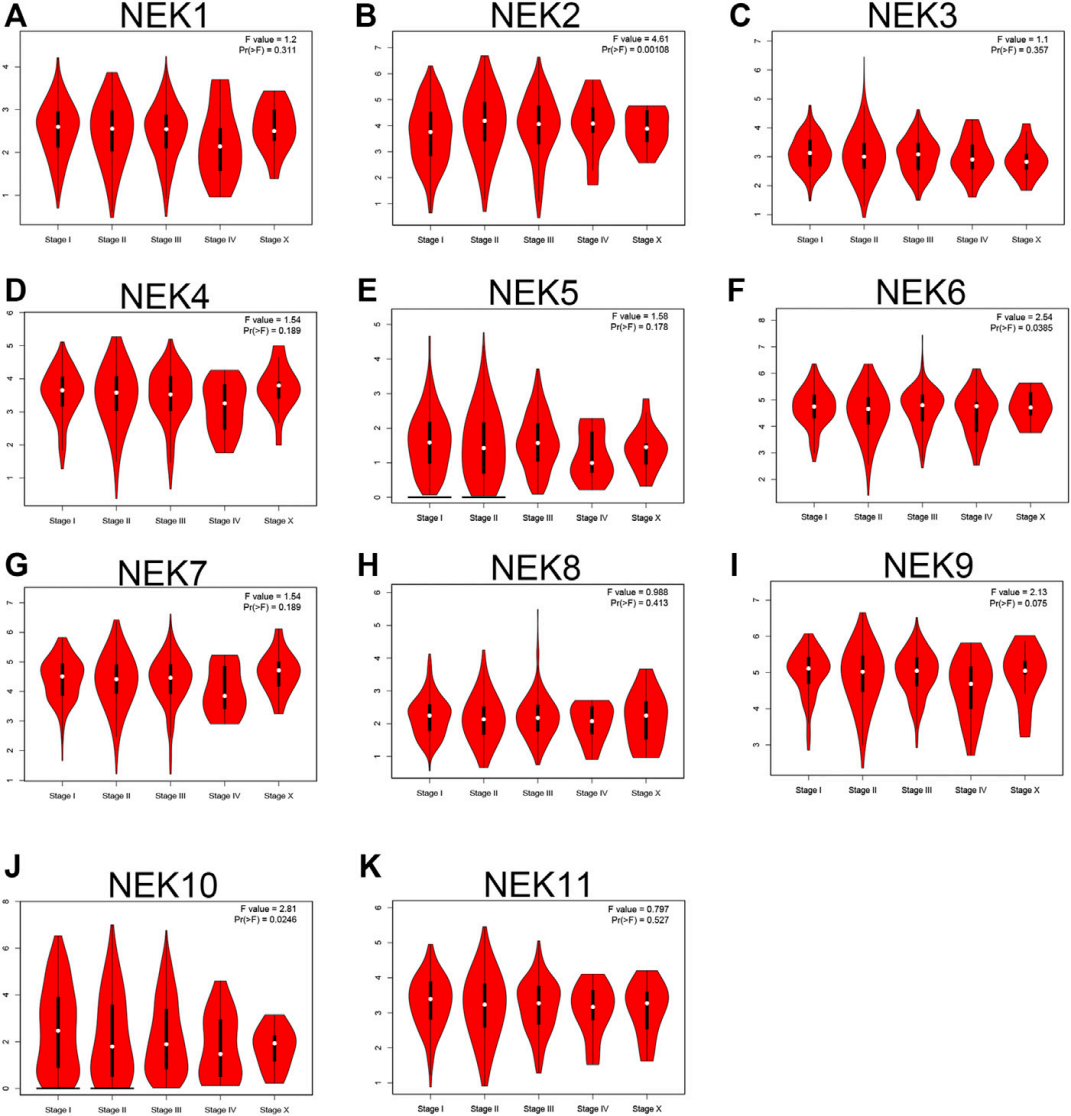
The correlation between the NEK family expression and clinical stage of BC patients. The graphs generated by using the GEPIA database show the expression of NEK family genes in different tumor stages of BC. **(A–K)** represents NEK family members NEK1–NEK11.

We further envisaged whether the transcriptional level of the NEK gene family was connected to the molecular subtypes and staging of BC after exploring the expression pattern of the NEK gene family in BC. To find out, we analyzed the data from the GEPIA2 and UALCAN databases, which are based on the TCGA database. As shown in Figure 5, most NEK gene families were highly expressed in luminal BC, including NEK1, NEK3, NEK4, NEK5, NEK7, NEK8, NEK9, NEK10, and NEK11. However, NEK6 was highly expressed in Her2+BC, and NEK2 was highly expressed in triple-negative BC. The expressions of NEK2, NEK6, and NEK10 were significantly correlated with BC (*p* < 0.05), while there was no difference in other NEK family genes (Figure 6).

### Role of the NIMA-Related Kinase Gene Family in the Prognosis of Breast Cancer Patients

**FIGURE 7:**
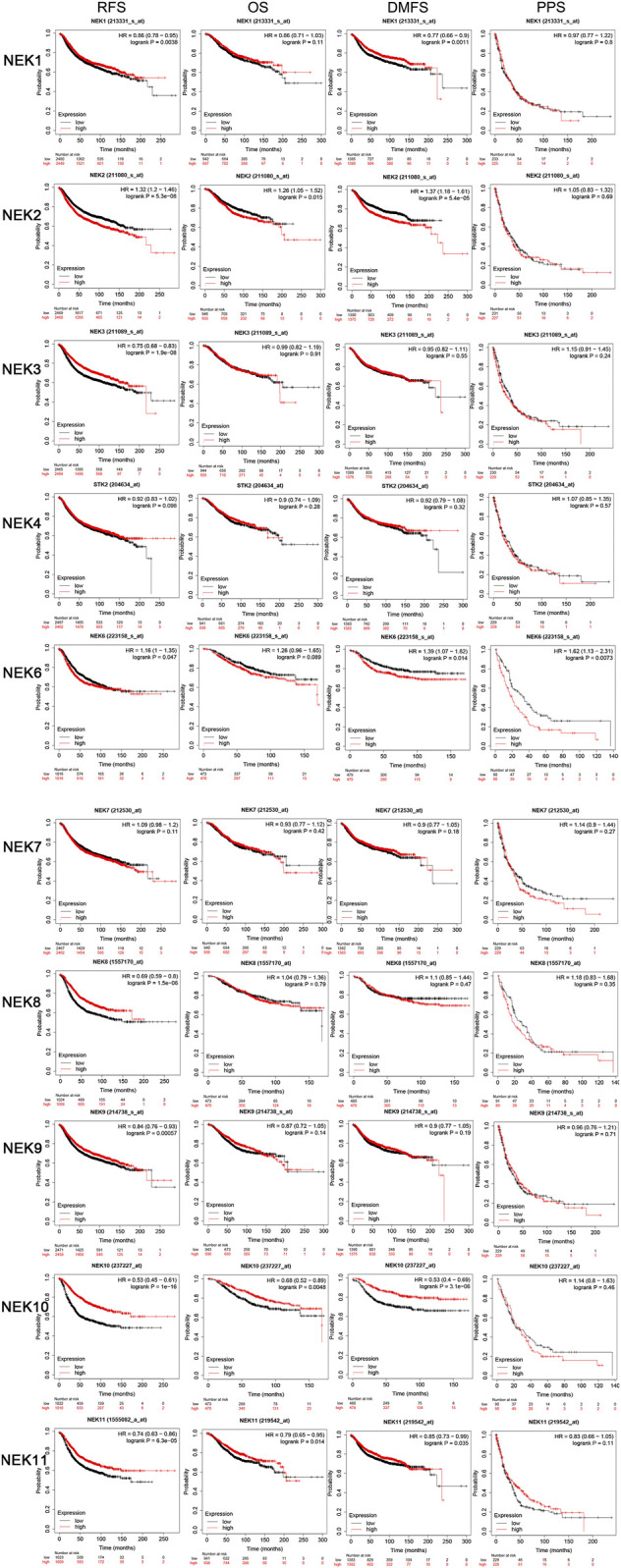
The prognostic significances of NEK family gene expression in BC patients. The curves generated by using the KM plotter database show the prognostic value of NEK family genes. The assessments include RFS, OS, DMFS, and PPS. The high expression is shown in red curves, and the low expression is shown in black curves. The log-rank test is displayed in the upper right corner of every graph.

In order to further understand the relationship about the expression level of tge NEK gene family with the OS, disease-free survival (DFS), distant metastasis-free survival (DMFS), and post-progression survival (PPS) in BC patients, the expression profile and clinical data of BC patients in the database were analyzed with the online Kaplan–Meier Plotter tool. As shown in Figure 7, high expression of NEK1, NEK3, NEK8, NEK9, NEK10, and NEK11 and low expression of NEK6 were positively correlated with the RFS of BC patients (*p* < 0.05). The high expression of NEK10 and NEK11 and low expression of NEK2 were positively correlated with the OS of BC patients (*p* < 0.05). The high expression of NEK1, NEK10, and NEK11 and low expression of NEK2 and NEK6 were positively correlated with the DMFS of BC patients (*p* < 0.05). Besides, the low expression of NEK6 was positively correlated with the PPS of BC patients (*p* < 0.05).

### Genetic Alterations of the NIMA-Related Kinase Gene Family in Breast Cancer

**FIGURE 8:**
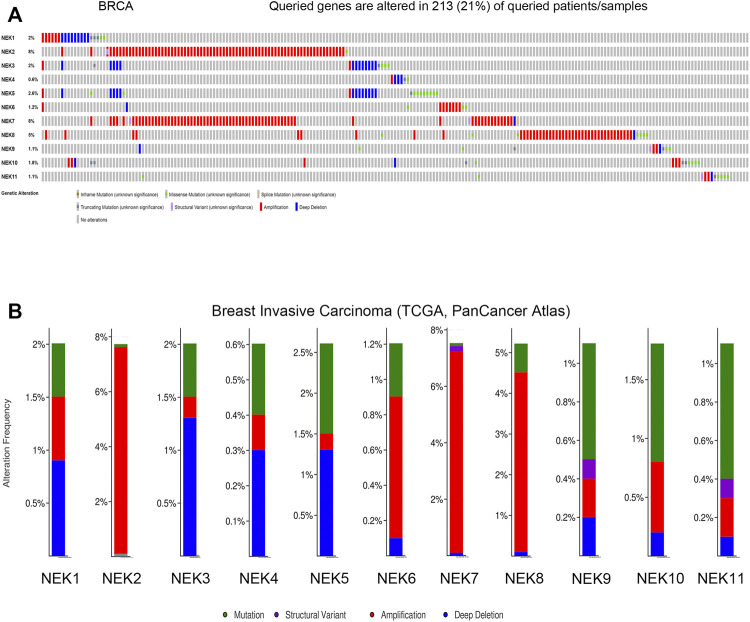
Genetic alterations and correlation analysis of NEK family members in BC. **(A)** summary of alteration rates for NEK family in BC (cBioPortal); **(B)** Genetic alteration frequency data of NEK family members in BC using cBioPortal.

The cBioPortal database was used to analyze the frequency and types of gene changes in the NEK gene family in 996 BC patients. As shown in Figure 8A, the variation rates of NEK 2 and NEK 7 genes were 8%, which is the highest in the NEK gene family. The variation rate of the NEK 4 gene was 0.4%, which is the lowest in the NEK gene family. In 996 BC samples, 213 patients had genetic variation in NEK genes, with a total variation rate of 33.4%. The main types of genetic variation in NEK family members include gene mutation, gene amplification, and gene-deep deletion. The mutation types of NEK2, NEK7, and NEK8 are mainly gene amplification (Figure 8B).

### Functional and Pathway Enrichment Analysis of NIMA-Related Kinase Gene Family-Related Molecules

**FIGURE 9:**
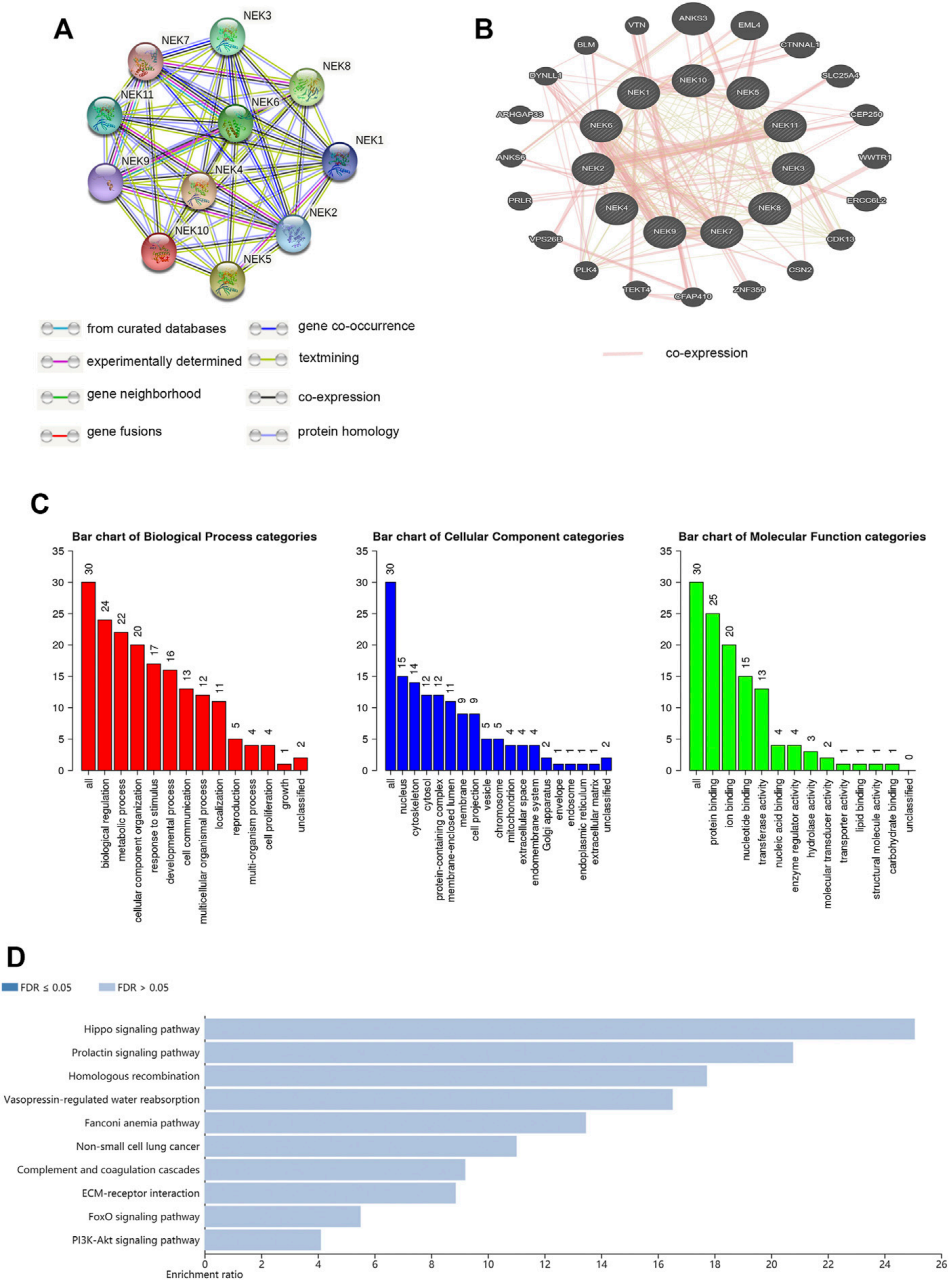
Predicted functions and pathways of the relevant molecules of the NEK gene family. **(A)** protein–protein interaction (PPI) of the 20 co-expressed genes of the NEK gene family in BC (STRING); **(B)** protein–protein interaction network of the NEK gene family (GeneMANIA); **(C)** GO functional enrichment analysis of relevant biological processes, cellular components, and molecular functions of the NEK gene family (WebGestalt); and **(D)** KEGG pathway analysis of the relevant signal pathways of the NEK gene family (WebGestalt).

The interaction between NEK gene family proteins was analyzed using the STRING 11.0 database. The NEK gene family was closely co-expressed (Figure 9A). We analyzed potential interaction partners with the NEK gene family with the WebGestalt database. ANKS3, EML4, CTNNAL1, SLC25A4, CEP250, WWTR1, ERCC6L2, CDK13, CSN2, ZNF350, CFAP410, TEKT4, PLK4, VSP26B, PRLR, ANKS6, ARHGAP33, DYNLL1, BLM, and VTN were closely associated with the NEK gene family by using the GeneMANIA website (Figure 9B). The WebGestalt database was used for GO and KEGG functional enrichment analysis of the above 20 genes related to the NEK gene family. GO analysis results showed that biological processes were concentrated in biological regulation, metabolic process, cellular component organization, and response to stimulus and developmental processes. The cell components were mainly concentrated in the nucleus, cytoskeleton, cytosol, protein-containing complex, and membrane-enclosed lumen. The molecular functional (MF) modules were mainly enriched in protein binding, ion binding, nucleotide binding, and transferase activity. The KEGG analysis results were mainly enriched in the hippo signaling pathway, prolactin signaling pathway, homologous recombination, vasopressin-regulated water reabsorption, and Fanconi anemia pathway.

### Correlation Between the NIMA-Related Kinase Gene Family and Tumor Immune Cell Infiltration

**FIGURE 10:**
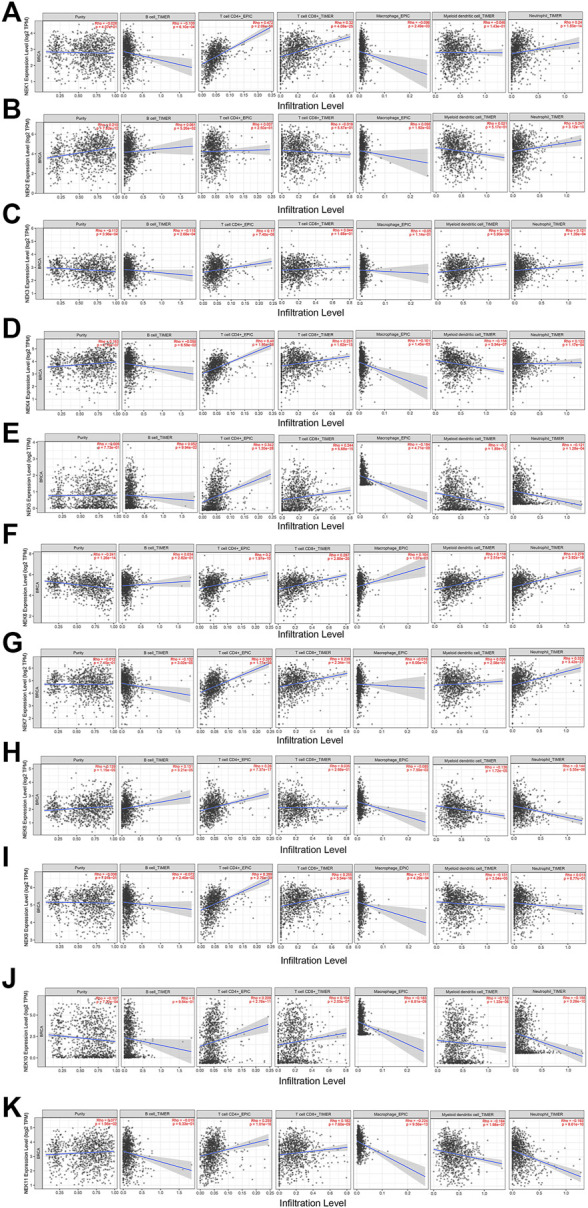
Correlation analysis between NEK gene family’s mRNAs and common immune cells. The expressions of the NEK gene family are significantly related to the immune infiltrating levels of B cells, CD8^+^ T cells, CD4+T cells, macrophages, neutrophils, and dendritic cells. *p* < 0.05 was considered statistically significant. **(A–K)** represents NEK family members NEK1–NEK11.

Immune cell infiltration is an integral part of the tumor microenvironment and an independent indicator of prognosis and lymph node metastasis. The correlation analysis of NEK and common immune cells in BC in the TIMER2.0 database is shown in Figure 9. Except for the expression level of NEK2, NEK3, and NEK8, the expression level of other NEK family genes were positively correlated with the number of CD8^+^ cells. Except for NEK2, the expression level of other NEK family genes were positively correlated with the number of CD4^+^ cells. The expression level of NEK8 was positively correlated with the number of B cells; the expression level of NEK1, NEK3, NEK4, NEK7, and NEK9 was negatively correlated with the number of B cells; the expression level of other NEK family genes was not correlated with the number of B cells. The expression level of NEK2, NEK3, NEK4, NEK6, and NEK7 was positively correlated with the number of neutrophils, while the expression level of NEK1, NEK5, NEK8, NEK10, and NEK11 was negatively correlated with the number of neutrophils. Except for NEK2, NEK6, and NEK7, the expression level of other NEK genes was negatively correlated with the number of macrophages. The expression level of NEK3 and NEK6 was positively correlated with the number of dendritic cells; the expression level of NEK4, NEK5, NEK8, NEK9, NEK10, and NEK11 was negatively correlated with the number of dendritic cells; the expression level of NEK1, NEK2, and NEK7 was not correlated with the number of dendritic cells (Figure 10, *p* < 0.05). These results indicated that the NEK gene family is important for regulating immune cells in BC.

## Discussion

With the rapid development of medicine, bioinformatics, as a new method to promote medical progress (Oulas et al., 2019), can quickly determine the key tumor treatment targets based on large sample sequencing data. BC has become one of the highest malignant tumors globally and poses great challenges to women’s health. Although predecessors have accumulated many research results, the molecular mechanism of its occurrence and development is still unclear. The prognosis of BC depends on its clinical diagnosis stage. It is significant to find markers related to the occurrence, development, and prognosis of BC and study its molecular mechanism. It is also necessary to explore more BC-related molecular targets to provide a new direction for the future treatment of BC.

The formation of the tumor is a process involving multiple genes, multiple pathways, and multiple molecules (Karki and Kanneganti, 2019), caused by the joint action of genetic and environmental factors. The abnormal expression of cell cycle kinases will cause acceleration of the cell cycle and over-proliferation, further leading to the occurrence of tumors (Malumbres and Barbacid, 2007).

The NEK was first discovered in the study of mitotic mutants of Aspergillus nidulans (Oakley and Morris, 1983). All 11 members in the NEK family have similar amino-catalytic region gene sequences with NIMA, which are different due to the length of the carboxyl terminal. NIMA-associated PKs are known as the third family of mitotic enzymes. Related enzymes in the NEK family, together with the Aurora family and PLK family, participate in and regulate a series of events after cyclin-dependent PK 1 activation during mitosis. The NEK family can regulate the formation and function of microtubules, which is related to the centrosome, and then affect the formation and function of cilia and spindle with microtubules as the main component and regulate the cell cycle. In recent years, NEK family genes have been found to be involved in the regulation of cell mitosis and have been called the third family of mitases. In many malignant tumors, abnormal expression of NEK protein will lead to abnormal regulation of the whole cell cycle, resulting in abnormal cell proliferation and the occurrence and development of tumors.

NEK2, NEK4, NEK5, NEK6, NEK8, and NEK11 were highly expressed in BC based on the UALCAN and GEPIA2 databases. High expression of these genes predicted a worse prognosis, suggesting that these genes could act as oncogenic genes in BC and novel biomarkers for predicting potential prognostic values and therapeutic targets of BC patients.

Many studies have shown that NEK2 is found in many human cancers, including non-small cell lung cancer (Zhong et al., 2014a; Zhong et al., 2014b), myeloma (Zhou et al., 2013), ovarian cancer (Liu et al., 2014), BC (Lee and Gollahon, 2013a; Lee and Gollahon, 2013b), prostate cancer (Zeng et al., 2015), colorectal cancer (Neal et al., 2014), malignant peripheral nerve sheath tumor (Stricker et al., 2013), and pancreatic ductal adenocarcinoma (Ning et al., 2014). The expression of NEK2 in tumor tissues was higher than that in healthy tissues. Moreover, Gangga Anuraga et al. revealed that NEK2 is involved in immune infiltration and may serve as a prognostic biomarker for breast cancer progression (Anuraga et al., 2021). Therefore, it may be considered that the increased expression of NEK2 in most human tumors is a common phenomenon. Besides, the abnormal expression of NEK2 will cause chromosome instability and aneuploidy, which are the most common phenomena in tumor cells. We preliminarily believed that high expression of NEK2 might be critical for the occurrence and development of tumors.

High expression of NEK3 usually predicts a poor prognosis of thyroid cancer (Melo-Hanchuk et al., 2020). NEK3 was highly expressed in normal breast tissues in this study, indicating a better RFS of BC. Ding et al. have demonstrated that NEK4 regulates EMT through Smads and ZEB1 to promote the occurrence and metastasis of lung cancer (Ding et al., 2018). In this study, NEK4 was highly expressed in BC tissues. The NEK5 gene has been reported to regulate prostate cancer (Nikitina et al., 2017) and BC (Pei et al., 2019). NEK5 promotes myogenic differentiation by up-regulating caspase-3 activity (Shimizu and Sawasaki, 2013). NEK5 is engaged in cell death and cell respiration (Melo Hanchuk et al., 2015). We found that NEK5 was highly expressed in BC tissues.

In recent years, NEK6 has been involved in the occurrence and development of many tumors, and its expression level has been significantly up-regulated in solid tumors (Jee et al., 2013). In this study, NEK6 was highly expressed in Her2+BC tissues and significantly correlated with the tumor stage, indicating poor RFS, DFS, and PPS of BC.

Sharif et al. have demonstrated that high expression of NEK7 is involved in the development of BC by activating NLRP3 inflammasomes (Sharif et al., 2019). We found that NEK7 was highly expressed in luminal BC tissues without the association with the stage and prognosis of BC.

Few studies have reported that NEK8 is related to the occurrence and development of tumors (Fry et al., 2012). Most of the relevant studies on NEK8 focus on its relationship with cystic nephropathy. Bowers et al. found that the expression of NEK8 in normal breast tissues was significantly lower than that in BC tissues (Bowers and Boylan et al., 2004). We found that NEK8 was highly expressed in luminal BC tissues, showing better a RFS of BC patients.

Studies have shown that NEK9 mediates the localization and recruitment of kinesin MKLP 2 and KIF14 related to cytokinesis (Cullati et al., 2017). In this study, NEK9 was highly expressed in luminal BC tissues, indicating a better RFS of BC.

NEK10 can promote the activation of MEK1, resulting in G2/M phase arrest and ERK1/2 phosphorylation. Knockdown of the human NEK10 gene can inhibit the phosphorylation of MEK1 and ERK1/2 (Moniz and Stambolic, 2011). We found that NEK10 was highly expressed in luminal BC tissues, significantly correlated with tumor stages, indicating a better RFS, OS, and DMFS of BC.

NEK11 is related to DNA damage response and DNA damage response checkpoint regulation in the G2/M phase (Noguchi et al., 2002). In this study, NEK11 was highly expressed in luminal BC tissues, showing a better RFS, OS, and DMFS of BC.

The occurrence and development of the tumor are complex in nature, including heterogeneous cellular components and the tumor immune microenvironment. Cell–cell interaction and cell–extracellular matrix interaction are important for different tumorigenesis, development, and metastasis stages. In this study, NEK2 and NEK7 had the highest mutation frequency in the NEK gene family. This study further expanded our understanding of the role of the NEK family in the occurrence and development of BC by conducting functional and pathway enrichment analysis of genes related to the NEK family in screened BC patients. The TIMER2.0 database analysis showed that NEK family gene expression was correlated with B cells, CD8 + T cells, CD4 + T cells, neutrophils, dendritic cells, and immune infiltration level of macrophages, suggesting that NEK family genes may participate in the immunity of BC cells.

This study also has some shortcomings. For example, we have not fully explained targeted drug screening related to the NEK family. The connectivity map (CMAP) is developed jointly by using the databases of Harvard University and the Massachusetts Institute of Technology. CMAP is a database that studies the chemical structure of drugs through the ratio of gene expression data, similar to finding drugs and their induction and the possible mechanism of drug molecules (Subramanian et al., 2017; Wang et al., 2020; Wang et al., 2021). In the future, we will perform relevant and targeted therapeutic drug screening based on different characteristics of NEK family members in this database.

In summary, based on the above open-access database and our experimental data, we revealed that not only NEK2 was highly expressed in breast cancer and correlated with prognosis, but also the expression level of NEK6, NEK8, and NEK11 were significantly correlated with molecular subtypes and prognosis of breast cancer, which are potential prognostic markers and new therapeutic targets. This study is the first to analyze the expression and prognosis of the NEK gene family in BC, laying a foundation for further research on the molecular mechanism of BC.

## Data Availability

The original contributions presented in the study are included in the article/Supplementary Material, further inquiries can be directed to the corresponding authors.

## References

[B1] Anuraga G. Wang W. J. Phan N. N. An Ton N. T. Ta H. D. K. Berenice Prayugo F. (2021). Potential Prognostic Biomarkers of NIMA (Never in Mitosis, Gene A)-Related Kinase (NEK) Family Members in Breast Cancer. J. Pers Med. 11 (11), 1089. 10.3390/jpm11111089 34834441 PMC8625415

[B2] Asplund A. Edqvist P.-H. D. Schwenk J. M. Pontén F. (2012). Antibodies for Profiling the Human Proteome-The Human Protein Atlas as a Resource for Cancer Research. Proteomics 12 (13), 2067–2077. 10.1002/pmic.201100504 22623277

[B3] Bowers A. J. Boylan J. F. (2004). Nek8, a NIMA Family Kinase Member, Is Overexpressed in Primary Human Breast Tumors. Gene 328, 135–142. 10.1016/j.gene.2003.12.002 15019993

[B4] Bray F. Ferlay J. Soerjomataram I. Siegel R. L. Torre L. A. Jemal A. (2018). Global Cancer Statistics 2018: GLOBOCAN Estimates of Incidence and Mortality Worldwide for 36 Cancers in 185 Countries. CA: A Cancer J. Clinicians 68 (6), 394–424. 10.3322/caac.21492 30207593

[B5] Chandrashekar D. S. Bashel B. Balasubramanya S. A. H. Creighton C. J. Ponce-Rodriguez I. Chakravarthi B. V. S. K. (2017). UALCAN: A Portal for Facilitating Tumor Subgroup Gene Expression and Survival Analyses. Neoplasia 19 (8), 649–658. 10.1016/j.neo.2017.05.002 28732212 PMC5516091

[B6] Cullati S. N. Kabeche L. Kettenbach A. N. Gerber S. A. (2017). A Bifurcated Signaling cascade of NIMA-Related Kinases Controls Distinct Kinesins in Anaphase. J. Cel Biol 216 (8), 2339–2354. 10.1083/jcb.201512055 PMC5551695 28630147

[B7] de Carcer G. Perez de Castro I. Malumbres M. (2007). Targeting Cell Cycle Kinases for Cancer Therapy. Cmc 14 (9), 969–985. 10.2174/092986707780362925 17439397

[B8] Ding N.-H. Zhang L. Xiao Z. Rong Z.-X. Li Z. He J. (2018). NEK4 Kinase Regulates EMT to Promote Lung Cancer Metastasis. J. Cel Mol Med 22 (12), 5877–5887. 10.1111/jcmm.13857 PMC6237562 30247800

[B9] Dou X.-W. Liang Y.-K. Lin H.-Y. Wei X.-L. Zhang Y.-Q. Bai J.-W. (2017). Notch3 Maintains Luminal Phenotype and Suppresses Tumorigenesis and Metastasis of Breast Cancer via Trans-activating Estrogen Receptor-α. Theranostics 7 (16), 4041–4056. 10.7150/thno.19989 29109797 PMC5667424

[B10] Franz M. Rodriguez H. Lopes C. Zuberi K. Montojo J. Bader G. D. (2018). GeneMANIA Update 2018. Nucleic Acids Res. 46 (W1), W60–W64. 10.1093/nar/gky311 29912392 PMC6030815

[B11] Fry A. M. O'Regan L. Sabir S. R. Bayliss R. (2012). Cell Cycle Regulation by the NEK Family of Protein Kinases. J. Cel Sci 125 (Pt 19), 4423–4433. 10.1242/jcs.111195 PMC3500863 23132929

[B12] Gao J. Aksoy B. A. Dogrusoz U. Dresdner G. Gross B. Sumer S. O. (2013). Integrative Analysis of Complex Cancer Genomics and Clinical Profiles Using the cBioPortal. Sci. Signal. 6 (269), pl1. 10.1126/scisignal.2004088 23550210 PMC4160307

[B13] Jee H. J. Kim H.-J. Kim A. J. Song N. Kim M. Lee H.-J. (2013). The Inhibition of Nek6 Function Sensitizes Human Cancer Cells to Premature Senescence upon Serum Reduction or Anticancer Drug Treatment. Cancer Lett. 335 (1), 175–182. 10.1016/j.canlet.2013.02.012 23416273

[B14] Joseph B. B. Wang Y. Edeen P. Lažetić V. Grant B. D. Fay D. S. (2020). Control of Clathrin-Mediated Endocytosis by NIMA Family Kinases. Plos Genet. 16 (2), e1008633. 10.1371/journal.pgen.1008633 32069276 PMC7048319

[B15] Karki R. Kanneganti T.-D. (2019). Diverging Inflammasome Signals in Tumorigenesis and Potential Targeting. Nat. Rev. Cancer 19 (4), 197–214. 10.1038/s41568-019-0123-y 30842595 PMC6953422

[B16] Lapenna S. Giordano A. (2009). Cell Cycle Kinases as Therapeutic Targets for Cancer. Nat. Rev. Drug Discov. 8 (7), 547–566. 10.1038/nrd2907 19568282

[B17] Lee J. Gollahon L. (2013). Mitotic Perturbations Induced by Nek2 Overexpression Require Interaction with TRF1 in Breast Cancer Cells. Cell Cycle 12 (23), 3599–3614. 10.4161/cc.26589 24091727 PMC3903712

[B18] Lee J. Gollahon L. (2013). Nek2-targeted ASO or siRNA Pretreatment Enhances Anticancer Drug Sensitivity in Triple-Negative Breast Cancer Cells. Int. J. Oncol. 42 (3), 839–847. 10.3892/ijo.2013.1788 23340795 PMC3597451

[B19] Liu X. Gao Y. Lu Y. Zhang J. Li L. Yin F. (2014). Upregulation of NEK2 Is Associated with Drug Resistance in Ovarian Cancer. Oncol. Rep. 31 (2), 745–754. 10.3892/or.2013.2910 24337664

[B20] Malumbres M. Barbacid M. (2007). Cell Cycle Kinases in Cancer. Curr. Opin. Genet. Dev. 17 (1), 60–65. 10.1016/j.gde.2006.12.008 17208431

[B21] Malumbres M. (2011). Physiological Relevance of Cell Cycle Kinases. Physiol. Rev. 91 (3), 973–1007. 10.1152/physrev.00025.2010 21742793

[B22] Melo Hanchuk T. D. Papa P. F. La Guardia P. G. Vercesi A. E. Kobarg J. (2015). Nek5 Interacts with Mitochondrial Proteins and Interferes Negatively in Mitochondrial Mediated Cell Death and Respiration. Cell Signal. 27 (6), 1168–1177. 10.1016/j.cellsig.2015.02.021 25725288

[B23] Melo-Hanchuk T. D. Martins M. B. Cunha L. L. Soares F. A. Ward L. S. Vassallo J. (2020). Expression of the NEK Family in normal and Cancer Tissue: an Immunohistochemical Study. BMC Cancer 20 (1), 23. 10.1186/s12885-019-6408-4 31906878 PMC6945616

[B24] Moniz L. S. Stambolic V. (2011). Nek10 Mediates G 2/M Cell Cycle Arrest and MEK Autoactivation in Response to UV Irradiation. Mol. Cel Biol 31 (1), 30–42. 10.1128/mcb.00648-10 PMC3019845 20956560

[B25] Neal C. P. Fry A. M. Moreman C. McGregor A. Garcea G. Berry D. P. (2014). Overexpression of the Nek2 Kinase in Colorectal Cancer Correlates with Beta-Catenin Relocalization and Shortened Cancer-specific Survival. J. Surg. Oncol. 110 (7), 828–838. 10.1002/jso.23717 25043295

[B26] Nikitina A. S. Sharova E. I. Danilenko S. A. Butusova T. B. Vasiliev A. O. Govorov A. V. (2017). Novel RNA Biomarkers of Prostate Cancer Revealed by RNA-Seq Analysis of Formalin-Fixed Samples Obtained from Russian Patients. Oncotarget 8 (20), 32990–33001. 10.18632/oncotarget.16518 28380430 PMC5464844

[B27] Ning Z. Wang A. Liang J. Liu J. Zhou T. Yan Q. (2014). Abnormal Expression of Nek2 in Pancreatic Ductal Adenocarcinoma: a Novel Marker for Prognosis. Int. J. Clin. Exp. Pathol. 7 (5), 2462–2469. 24966957 PMC4069945

[B28] Noguchi K. Fukazawa H. Murakami Y. Uehara Y. (2002). Nek11, a New Member of the NIMA Family of Kinases, Involved in DNA Replication and Genotoxic Stress Responses. J. Biol. Chem. 277 (42), 39655–39665. 10.1074/jbc.m204599200 12154088

[B29] Oakley B. Morris R. (1983). A Mutation in Aspergillus nidulans that Blocks the Transition from Interphase to Prophase. J. Cel Biol 96 (4), 1155–1158. 10.1083/jcb.96.4.1155 PMC2112314 6339527

[B30] Oulas A. Minadakis G. Zachariou M. Sokratous K. Bourdakou M. M. Spyrou G. M. (2019). Systems Bioinformatics: Increasing Precision of Computational Diagnostics and Therapeutics through Network-Based Approaches. Brief Bioinform 20 (3), 806–824. 10.1093/bib/bbx151 29186305 PMC6585387

[B31] Pei J. Zhang J. Yang X. Wu Z. Sun C. Wang Z. (2019). NEK5 Promotes Breast Cancer Cell Proliferation through Up‐regulation of Cyclin A2. Mol. Carcinog 58 (6), 933–943. 10.1002/mc.22982 30675923

[B32] Peres de Oliveira A. Basei F. L. Slepicka P. F. de Castro Ferezin C. Melo-Hanchuk T. D. de Souza E. E. (2020). NEK10 Interactome and Depletion Reveal New Roles in Mitochondria. Proteome Sci. 18, 4. 10.1186/s12953-020-00160-w 32368190 PMC7189645

[B33] Prosser S. L. O'Regan L. Fry A. M. (2016). Novel Insights into the Mechanisms of Mitotic Spindle Assembly by NEK Kinases. Mol. Cell Oncol. 3 (3), e1062952. 10.1080/23723556.2015.1062952 27314078 PMC4909405

[B34] Sharif H. Wang L. Wang W. L. Magupalli V. G. Andreeva L. Qiao Q. (2019). Structural Mechanism for NEK7-Licensed Activation of NLRP3 Inflammasome. Nature 570 (7761), 338–343. 10.1038/s41586-019-1295-z 31189953 PMC6774351

[B35] Shimizu K. Sawasaki T. (2013). Nek5, a Novel Substrate for Caspase-3, Promotes Skeletal Muscle Differentiation by Up-Regulating Caspase Activity. FEBS Lett. 587 (14), 2219–2225. 10.1016/j.febslet.2013.05.049 23727203

[B36] Stricker T. P. Henriksen K. J. Tonsgard J. H. Montag A. G. Krausz T. N. Pytel P. (2013). Expression Profiling of 519 Kinase Genes in Matched Malignant Peripheral Nerve Sheath Tumor/plexiform Neurofibroma Samples Is Discriminatory and Identifies Mitotic Regulators BUB1B, PBK and NEK2 as Overexpressed with Transformation. Mod. Pathol. 26 (7), 930–943. 10.1038/modpathol.2012.242 23370767

[B37] Subramanian A. Narayan R. Corsello S. M. Peck D. D. Natoli T. E. Lu X. (2017). A Next Generation Connectivity Map: L1000 Platform and the First 1,000,000 Profiles. Cell 171 (6), 1437–e17. 10.1016/j.cell.2017.10.049 29195078 PMC5990023

[B38] Sun C.-C. Li S.-J. Hu W. Zhang J. Zhou Q. Liu C. (2019). Comprehensive Analysis of the Expression and Prognosis for E2Fs in Human Breast Cancer. Mol. Ther. 27 (6), 1153–1165. 10.1016/j.ymthe.2019.03.019 31010740 PMC6554685

[B39] Szklarczyk D. Gable A. L. Nastou K. C. Lyon D. Kirsch R. Pyysalo S. (2021). The STRING Database in 2021: Customizable Protein-Protein Networks, and Functional Characterization of User-Uploaded Gene/measurement Sets. Nucleic Acids Res. 49 (D1), D605–D612. 10.1093/nar/gkaa1074 33237311 PMC7779004

[B40] Wang C.-Y. Chao Y.-J. Chen Y.-L. Wang T.-W. Phan N. N. Hsu H.-P. (2021). Upregulation of Peroxisome Proliferator-Activated Receptor-α and the Lipid Metabolism Pathway Promotes Carcinogenesis of Ampullary Cancer. Int. J. Med. Sci. 18 (1), 256–269. 10.7150/ijms.48123 33390794 PMC7738964

[B41] Wang C. Y. Chiao C. C. Phan N. N. Li C. Y. Sun Z. D. Jiang J. Z. (2020). Gene Signatures and Potential Therapeutic Targets of Amino Acid Metabolism in Estrogen Receptor-Positive Breast Cancer. Am. J. Cancer Res. 10 (1), 95–113. 32064155 PMC7017735

[B42] Warde-Farley D. Donaldson S. L. Comes O. Zuberi K. Badrawi R. Chao P. (2010). The GeneMANIA Prediction Server: Biological Network Integration for Gene Prioritization and Predicting Gene Function. Nucleic Acids Res. 38, W214–W220. 10.1093/nar/gkq537 20576703 PMC2896186

[B43] Zeng Y.-R. Han Z.-D. Wang C. Cai C. Huang Y.-Q. Luo H.-W. (2015). Overexpression of NIMA-Related Kinase 2 Is Associated with Progression and Poor Prognosis of Prostate Cancer. BMC Urol. 15, 90. 10.1186/s12894-015-0085-7 26320076 PMC4553013

[B44] Zhong X. Guan X. Dong Q. Yang S. Liu W. Zhang L. (2014). Examining Nek2 as a Better Proliferation Marker in Non-small Cell Lung Cancer Prognosis. Tumor Biol. 35 (7), 7155–7162. 10.1007/s13277-014-1935-8 24763826

[B45] Zhong X. Guan X. Liu W. Zhang L. (2014). Aberrant Expression of NEK2 and its Clinical Significance in Non-small Cell Lung Cancer. Oncol. Lett. 8 (4), 1470–1476. 10.3892/ol.2014.2396 25202351 PMC4156209

[B46] Zhou W. Yang Y. Xia J. Wang H. Salama M. E. Xiong W. (2013). NEK2 Induces Drug Resistance Mainly through Activation of Efflux Drug Pumps and Is Associated with Poor Prognosis in Myeloma and Other Cancers. Cancer Cell 23 (1), 48–62. 10.1016/j.ccr.2012.12.001 23328480 PMC3954609

